# Intraspecific macroscopic digestive anatomy of ring-tailed lemurs (*Lemur catta*), including a comparison of frozen and formalin-stored specimens

**DOI:** 10.1007/s10329-020-00873-8

**Published:** 2020-11-12

**Authors:** Marcus Clauss, Jelscha Trümpler, Nicole L. Ackermans, Andrew C. Kitchener, Georg Hantke, Julia Stagegaard, Tomo Takano, Yuta Shintaku, Ikki Matsuda

**Affiliations:** 1 Clinic for Zoo Animals, Exotic Pets and Wildlife, Vetsuisse Faculty, University of Zurich, Winterthurerstrasse 260, 8057 Zurich, Switzerland; 2 Department of Natural Sciences, National Museums Scotland, Chambers Street, Edinburgh, EH1 1JF UK; 3 Ree Park Safari, Stubbe Soevej 15, 8400 Ebeltoft, Denmark; 4 Japan Monkey Centre, Inuyama, Japan; 5 Chubu University Academy of Emerging Sciences, 1200, Matsumoto-cho, Kasugai, Aichi 487-8501 Japan; 6 Wildlife Research Center, Kyoto University, Kyoto, Japan; 7 Institute for Tropical Biology and Conservation, Universiti Malaysia Sabah, Kota Kinabalu, Sabah Malaysia; 8 Present Address: Center for Anatomy and Functional Morphology, Icahn School of Medicine at Mount Sinai, Annenberg Building, 1468 Madison Avenue, New York, NY 10029 USA

**Keywords:** Anatomy, Allometry, Digestive tract, Primates, Strepsirrhini

## Abstract

Digestive tract measurements are often considered species specific, but little information exists on the degree to which they change during ontogeny within a species. Additionally, access to anatomical material from nondomestic species is often limited, with fixed tissues possibly representing the only available source, though the degree to which this material is representative in terms of dimensions and weight is debatable. In the present study, the macroscopic anatomy of the digestive tract (length of intestinal sections, and tissue weights of stomach and intestines) of 58 *Lemur catta* [ranging in age from 1 month (neonates) to 25 years], which had been stored frozen (*n* = 27) or fixed in formalin (*n* = 31), was quantified. Particular attention was paid to the caecum and the possible presence of an appendix. The intraspecific allometric scaling of body mass (BM)^0.46[0.40;0.51]^ for total intestine length and BM^0.48[0.41;0.54]^ for small intestine length was higher than the expected geometric scaling of BM^0.33^, and similar to that reported in the literature for interspecific scaling. This difference in scaling is usually explained by the hypothesis that, to maintain optimal absorption, the diameter of the intestinal tube cannot increase geometrically. Therefore, geometric volume gain of increasing body mass is accommodated for by more-than-geometric length scaling. According to the literature, not all *L. catta* have an appendix. No appendix was found in the specimens in the present study. The proportions of length measurements did not change markedly during ontogeny, indicating that the proportions of the foetus are representative of those of the adult animal. By contrast, width and tissue-mass scaling of the caecum indicated disproportionate growth of this organ during ontogeny that was not reflected in its length. Compared to overall intraspecific variation, the method of storage (frozen vs. formalin) had no relevant impact on length or weight measurements.

**Electronic supplementary material:**

The online version of this article (10.1007/s10329-020-00873-8) contains supplementary material, which is available to authorized users.

## Introduction

Based on geometric considerations, volume measurements should scale isometrically (in other words, linearly) with body mass, surface measurements should scale to body mass to the power of 0.67, and length measurements—such as those of intestinal tract sections—should scale to body mass to the power of 0.33 (Calder 1996; Clauss and Hummel 2005). Scaling relationships that are not isometric (or linear) are typically called ‘allometric’ (verbatim translation: ‘another measure’), and there is a certain tradition to equate ‘allometric scaling’ with ‘geometric scaling,’ so that ‘positive allometry’ indicates a higher exponent than expected due to geometric rules.

In contrast to the expected geometric scaling of length measurements, several studies found a higher scaling exponent (positive allometry) for interspecific scaling relationships of various intestinal section lengths with body mass in mammals (Woodall and Skinner 1993; Lavin et al. 2008; McGrosky et al. 2016, 2019a, b). The common explanation for this observation, developed to our knowledge by Woodall and Skinner (1993), is that on the one hand, both intestinal volume and surface area (calculated from length and circumference measurements in their study) do indeed scale geometrically with body mass, but that intestinal diameter scales to a lower exponent in order to maintain short diffusion distances from the lumen to the secretory and absorptive surfaces. Therefore, the length of the intestine must scale more-than-geometrically (with positive allometry) to accommodate geometric volume and surface scaling. If this reasoning were correct, we would expect similar scaling at the intraspecific level across ontogeny, particularly because the transition from milk to any other diet likely implies a decrease in diet digestibility, theoretically making short distances between the lumen and surface all the more relevant.

On a completely different level of consideration, anatomical material from nondomestic species can be hard to come by, but data derived from it are important for comparative studies with a physiological as well as an evolutionary focus (e.g., Lavin et al. 2008; Smith et al. 2017). Historically, macroanatomical studies often relied on comparatively older literature [Crile and Quiring (1940); cf. the instructive example given by Ridgway and Van Alstyne (2017)]. Given that hunting expeditions or culling operations are no longer socially acceptable, the accumulation of samples typically depends on the storage of deceased individuals collected as single specimens from zoological collections, or during fieldwork. Typically, these specimens are either stored frozen or fixed in formalin. Compared to frozen storage of fresh material, storage in formalin often leads to tissue shrinkage (Lentle et al. 1997); comparisons of intestine length measurements between frozen and formalin-fixed material of a limited number of specimens indicated some degree of shortening during formalin storage (Hume et al. 1993). More recently, a comparative study in humans, in which intestine length was measured during abdominal surgery in live patients and at dissection in formalin-fixed cadavers, indicated significantly shorter intestine length for the latter; the length measures for the fixed specimens were also shorter than those reported for freshly dissected cadavers in the literature (Zhou et al. 2020). Evidently, published results allow for the possibility that shrinkage occurs after death, irrespective of the method of storage used. For example, studies on skin samples indicated that tissue shrinkage occurred as an effect of excision and was not exacerbated by formalin storage (Dauendorffer et al. 2009), and Parker (1963) found that fish shrank shortly after killing, irrespective of the preservation method, without additional effects of longer storage. For intestines, both the effects of relaxation–elongation and of contraction–shortening after death have been reported in the literature (Zhou et al. 2020). An older comprehensive study in dogs documented that intestinal shortening of fresh material occurred within the first few hours after death (Nickel 1933). This shortening sometimes persisted, but was more often followed by relaxation that exceeded the effects of shortening within 48 h, leading to longer-than-life measurements at this timepoint that were considered to represent the relaxation of the natural tonus of the smooth intestinal musculature (Nickel 1933). These findings add to the overall uncertainty concerning intestinal length measurements, and indicate that the effects of storage and fixation depend on the state of the material at the moment of fixation or freezing. There are most likely many other factors, such as whether material is frozen or fixed with or without the mesenteries, the temperature at dissection, or the forces involved in laying out an intestinal section in a straight line for measurement (Underhill 1955), that could also influence the final outcome.

In the present study, we used the opportunity to access three different collections of gastrointestinal tracts of ring-tailed lemurs (*Lemur catta*), either preserved frozen attached to the mesenteries, or preserved in formalin after dissection of the mesenteries. The main aims of the study were to test whether intraspecific allometries of intestinal lengths resembled those reported for interspecific comparisons in other mammals, and whether a systematic difference between the two preservation methods could be detected. In addition, we aimed to investigate whether the prominence of the caecum, the site of microbial fermentation of complex carbohydrates derived from plant fibre (Campbell et al. 2000), changed with ontogeny from milk-dependent neonates to mature individuals. Ring-tailed lemurs have been described as a species in which a caecal appendix may occur variably (Smith et al. 2013, 2017), although macroscopic descriptions of the caecum did not indicate the presence of an appendix (Campbell et al. 2000; McGrosky et al. 2019b). Therefore, special attention was given to the appearance of the apex of the caecum.

## Methods

**Fig. 1:**
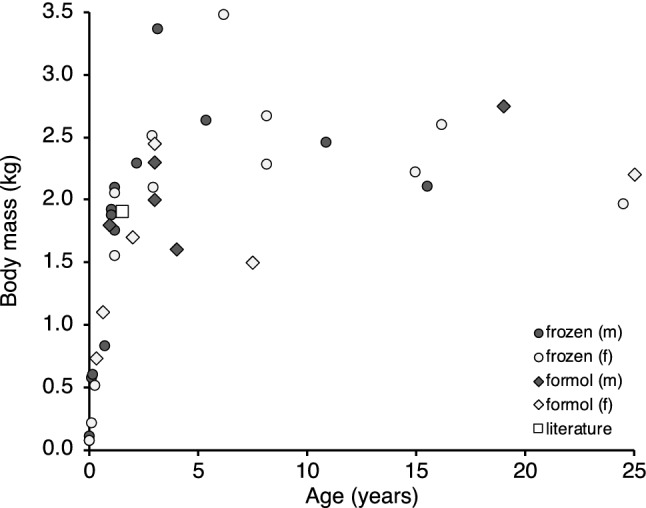
Relationship of body mass and age in male (*m*) and female (*f*) ring-tailed lemurs (*Lemur catta*) for which age was known in the present study, and for which the intestine was stored frozen or in formalin (*formal*). Note two particularly heavy animals with a body mass > 3 kg, which were excluded from subsequent analyses

Three different collections of ring-tailed lemur specimens (*Lemur catta*; *n* = 58) were available for this study (Table 1). The first consisted of 12 specimens from various zoological collections, stored frozen as whole carcasses for varying amounts of time, and thawed and dissected for the present study. The second set consisted of 15 specimens of a large family group originating from a single zoological facility; the gastrointestinal tract of each animal, including all mesenteries, had been excised immediately after death and stored frozen (for 12 months) until dissection. The third set consisted of 31 specimens from a single zoological collection, where the gastrointestinal tract had been dissected from freshly deceased specimens, freed of mesenteries to varying degrees, partially opened (lengthwise), and stored in formalin for varying amounts of time. The body mass of 18 of the animals surpassed the range of mean body mass, 2–2.5 kg, reported for adult free-living ring-tailed lemurs (Sussman 1991; Drea and Weil 2008), yet our age–body mass graph (Fig. 1) largely resembled that given by Koyama et al. (2008; adult body masses between 2 and 3 kg), and only two animals appeared to be of excessive weight for their age, being distinctively heavier than the maximum weight, 2.6 kg, recorded for ring-tailed lemurs in their natural habitat by Simmen et al. (2010). These findings suggested that obesity was not a major factor in the study populations, and only the two heaviest animals were excluded from the analyses.

**Table 1:** Overview of the three sets of gastrointestinal tracts (*GIT*) of *Lemur catta* used in the present study

Collection	Origin	*n* (females, males)	Age range	Body mass range (kg)	Preservation method
A	Various zoological collections	12 (7, 5)	Neonate-24 years	0.07–3.48	Whole carcasses, stored frozen (for 3–20 years), thawed and dissected for the present study
B	Single zoological collection	15 (6, 9)	0.1–16 years	0.57–2.67	GIT, including mesenteries, dissected directly after death, stored frozen for 1 year, thawed and prepared for the present study
C	Single zoological collection	31 (19, 12)	0.33–25 years	0.73–2.85	GIT dissected after death, freed of mesenteries, partially opened, stored in formalin (for 1–60 years)

**Fig. 2:**
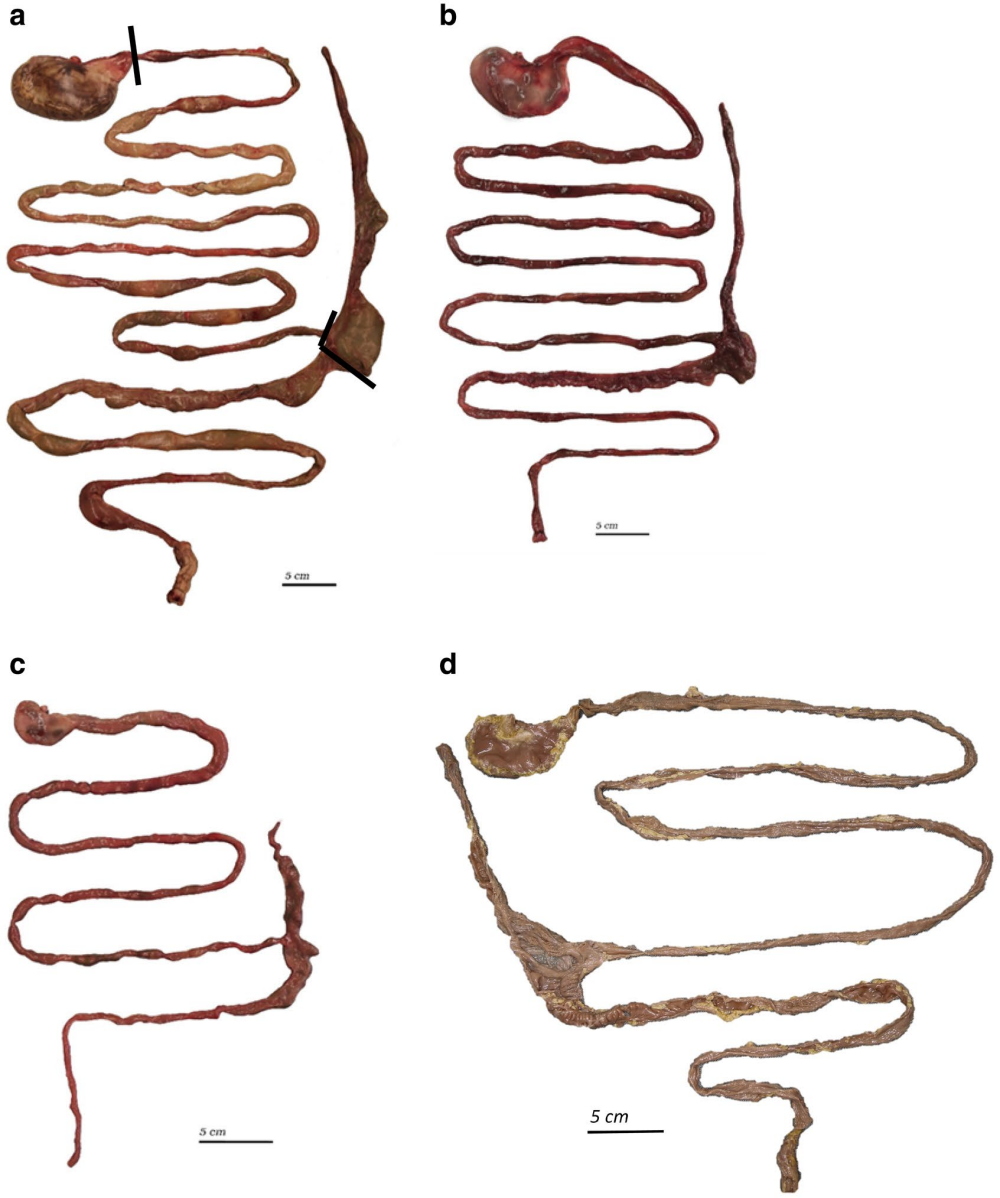
Gastrointestinal tracts (GIT) of various ring-tailed lemurs (*L. catta*) stored frozen (**a**–**c**) or in formalin (**d**): 16.2-year-old female, body mass 2.6 kg (**a**); 8.2-year-old female, body mass 2.3 kg (**b**); 0.1-year-old male, body mass 0.6 kg (**c**); adult female, body mass 2.1 kg (**d**). **a** Measurement borders for the small intestine, caecum and colon indicated by *black lines*

All gastrointestinal tracts were freed from mesenteries and adhering adipose tissue and photographed (Fig. 2). For photography and measurements, thawed intestines were laid out without deliberate stretching beyond that countered by the friction between the intestine and the metal dissection table. Intestines preserved in formalin were gently straightened for length measurements. Length measurements included those of the small intestine, the caecum, and the colon and rectum combined. In thawed (unopened), but not in formalin-preserved (and generally opened) caeca, the width at the base was measured as well. Because of its non-tubular structure and the corresponding greater difficulty in defining a line of measurement, the length of the stomach was not measured. Subsequently, the stomach, small intestine, caecum and the joined colon–rectum were cleared of contents, blotted dry with paper towels, and weighed.

Statistical evaluations were performed in R (R Core Team 2017). Linear models based on log-transformed data were used. First, we tested the allometric relationships of all intestine lengths, caecum width and weights with body mass. Additionally, the effect of body mass on organ measurements expressed as percentage of either total intestinal length or total gastrointestinal tract (GIT) tissue weight was assessed in the same manner, to test whether changes in the prominence of organs occurred with maturation. These results are given to facilitate comparison with other allometries, even though residuals of the models were mostly not normally distributed. Then, the linear models were repeated using ranked data for quantitative intestine measures and body mass (making the models non-parametric), with the additional co-factors sex and preservation method (frozen or fixed) and their interaction. Whether body mass was a significant covariable in these secondary models or not was identical to whether the scaling exponent was significant in the primary models, and is therefore not indicated separately. The significance level was set to 0.05. For graphical representation, untransformed data are shown.

## Results

**Fig. 3:**
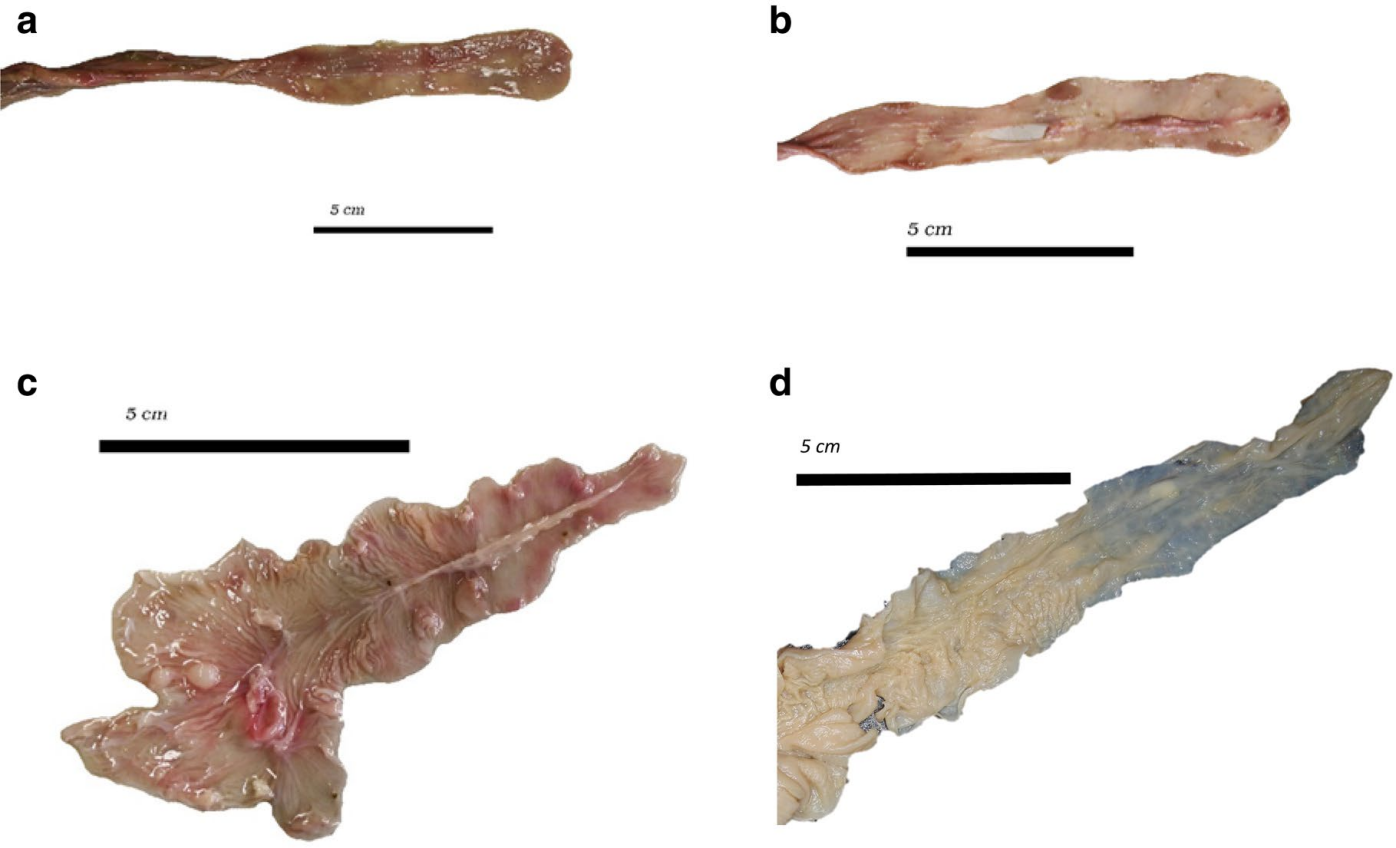
The opened caecum (apex pointing towards the* right*) of various ring-tailed lemurs (*L. catta*) stored frozen (**a**–**c**) or in formalin (**d**) indicating the absence of a caecal appendix: 16.2-year-old female, body mass 2.6 kg (see Fig. 2a) (**a**); 3.2-year-old male, body mass 3.4 kg (**b**); 0.1-year-old male, body mass 0.6 kg (see Fig. 2c) (**c**); adult female, body mass 1.5 kg (**d**)

The macroscopic appearance of the ring-tailed lemur digestive tract in the present study resembled that described by Campbell et al. (2000) and McGrosky et al. (2019b), with a simple stomach, an elongated and haustrated caecum, and a proximal colon with some haustration (Fig. 2). Although inspection of the caecum indicated that some individuals might have a caecal appendix (e.g., Fig. 2c), when the apex of the caecum was opened, no different section could not be discerned that resembled a lymphatic organ (Fig. 3).

**Fig. 4:**
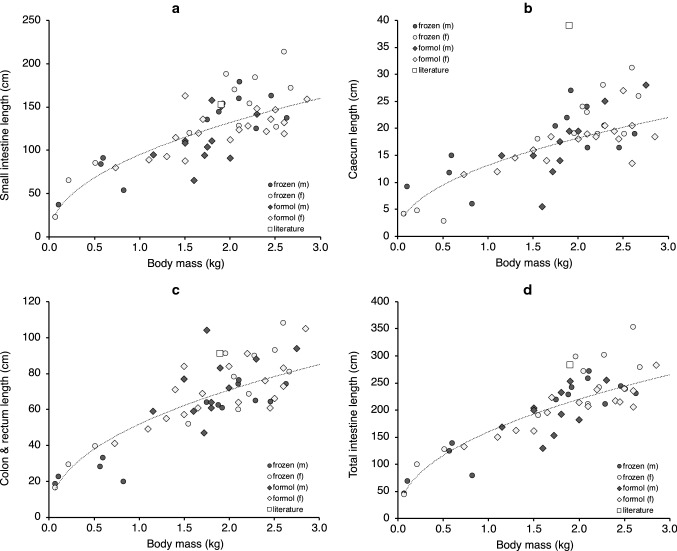
Relationship between body mass and **a** small intestine length, **b** caecum length, **c** colon and rectum length, and **d** total intestine length in ring-tailed lemurs (*L. catta*) in the present study (males and females, preserved frozen or in formalin) and in an individual reported by Campbell et al. (2000) (*literature*). For statistical analysis, see Table 2

**Fig. 5:**
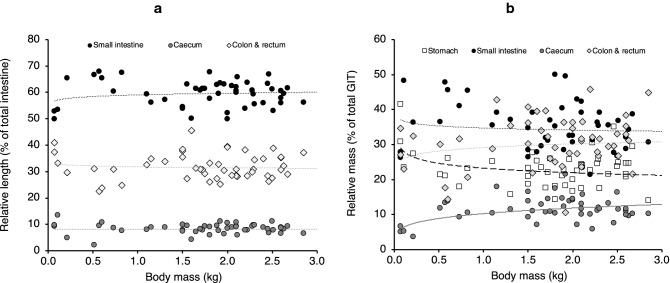
Relationship of body mass and **a** relative intestine lengths, **b** relative gastrointestinal organ masses (both in % of total) of ring-tailed lemurs (*L. catta*) in the present study. The only significant relationships are those for the relative mass of stomach and caecum (cf. Table 3)

The measurements taken in the present study were within a range that included those of an individual published by Campbell et al. (2000) (Fig. 4) except for the caecum, for which these authors reported a greater length. The allometric scaling of length measures of all intestinal sections yielded exponents with 95% confidence intervals above the 0.33 scaling exponent expected from geometry (i.e., there was positive allometry) (Table 2). The scaling exponent of caecum width was particularly high at 0.57. The relative length of the intestinal sections did not change with body mass, suggesting that their proportions (small intestine 59%, caecum 8%, colon and rectum 32% of the total intestinal length) remain stable during ontogeny (Table 2; Fig. 5a). Preservation method only had an effect on the length of the colon and rectum, which were longer for specimens preserved in formalin, but the results also indicated an interaction that suggested that the effect of preservation method differed according to sex (Table 2; Fig. 4c). Samples fixed in formalin had a slightly longer relative colon and rectum length (34 ± 4% vs. 30 ± 4%) and a shorter relative small intestine length (57 ± 4% vs. 62 ± 5%) (Table 2).

**Table 2:** Allometric regressions (*y* = *a* body mass^*b*^) and 95% confidence intervals (*CI*) for measures of intestinal lengths of ring-tailed lemurs (*L. catta*) according to sex and preservation method

Dependent variable (*y*)	*a* (95% CI)	*P*	*b* (95% CI)	*P*	Sex^a^	Preservation^a^	Interaction^a^
Absolute length (cm)							
Small intestine	95 (89, 101)	< 0.001	0.48 (0.41, 0.54)	< 0.001	n.s.	n.s.	n.s.
Caecum^b^	13 (12, 14)	< 0.001	0.47 (0.37, 0.58)	< 0.001	n.s.	n.s.	n.s.
Colon and rectum^b^	52 (49, 55)	< 0.001	0.45 (0.38, 0.52)	< 0.001	(+) 0.033	(+) 0.006	0.028
Total intestine^b^	160 (152, 168)	< 0.001	0.46 (0.40, 0.51)	< 0.001	n.s.	n.s.	n.s.
Caecum width	2.1 (1.9, 2.4)	< 0.001	0.57 (0.47, 0.67)	< 0.001	n.s.	–	–
Relative length (% total intestinal length)							
Small intestine	59 (58, 61)	< 0.001	0.01 (− 0.01, 0.04)	0.282	n.s.	n.s.	n.s.
Caecum^b^	8 (8, 9)	< 0.001	0.01 (− 0.09, 0.10)	0.913	n.s.	n.s.	n.s.
Colon and rectum	32 (30, 33)	< 0.001	− 0.01 (− 0.06, 0.03)	0.568	n.s.	(+) 0.014	n.s.

Body mass in kilograms.* Plus sign in parentheses* indicates higher values in females, or higher values in formalin-preserved tissues

*n.s.* Not significant

^a^Results of additional models testing for effects of sex and preservation using ranked data

^b^Residuals were not normally distributed

The body–mass scaling exponents for organ masses exceeded an isometric (or linear) scaling in their 95% confidence intervals, except for the stomach (Table 3). Of the intestine sections, the small intestine showed the least distinct deviation (exponent confidence interval 1.01; 1.28), and the caecum (1.21; 1.54) the most distinct deviation from isometry (linearity). As for length measures, preservation status had an effect on caecum and colon and rectum tissue mass (Table 3). Therefore, total intestinal mass and total GIT mass were slightly higher in formalin-fixed specimens (Table 3). Considering the entire GIT, the relative mass of the small intestine and colon & rectum did not vary with body mass; by contrast, the relative mass of the stomach declined, and that of the caecum increased, with body mass (Table 3; Fig. 5b). Considering only the intestinal tract, relative caecum mass increased with body mass (Table 3). Relative tissue mass of the colon and rectum of the total GIT was higher in formalin-fixed specimens (frozen: 27 ± 7%; fixed 35 ± 6% of total GIT mass) (Table 3).

**Table 3:** Allometric regressions for weight measurements (*y* = *a* body mass^*b*^) including 95% confidence intervals for tissue mass of GIT of ring-tailed lemurs (*L. catta*) according to sex and preservation method

Dependent variable (*y*)	*a* (95% CI)	*P*	*b* (95% CI)	*P*	Sex^a^	Preservation^a^	Interaction^a^
Absolute mass (g)							
Stomach^b^	6.9 (6.2, 7.8)	< 0.001	1.08 (0.95, 1.20)	< 0.001	n.s.	n.s.	n.s.
Small intestine^b^	10.5 (9.3, 11.9)	< 0.001	1.14 (1.01, 1.28)	< 0.001	n.s.	n.s.	n.s.
Caecum^b^	3.0 (2.6, 3.6)	< 0.001	1.37 (1.21, 1.54)	< 0.001	n.s.	(+) 0.048	n.s.
Colon and rectum^b^	9.0 (7.7, 10.6)	< 0.001	1.22 (1.05, 1.39)	< 0.001	n.s.	(+) 0.002	n.s.
Total intestine^b^	22.8 (20.1, 25.9)	< 0.001	1.20 (1.06, 1.33)	< 0.001	n.s.	(+) 0.017	n.s.
Total GIT^b^	29.8 (26.4, 33.7)	< 0.001	1.17 (1.04, 1.30)	< 0.001	n.s.	(+) 0.027	n.s.
Relative mass (% total GIT mass)							
Stomach	23 (22, 25)	< 0.001	− 0.08 (− 0.15, − 0.02)	0.014	n.s.	n.s.	n.s.
Small intestine	34 (33, 37)	< 0.001	− 0.02 (− 0.09, 0.04)	0.458	n.s.	n.s.	n.s.
Caecum^c^	10 (9, 11)	< 0.001	0.21 (0.12, 0.30)	< 0.001	n.s.	n.s.	n.s.
Colon and rectum	29 (27, 32)	< 0.001	0.05 (− 0.03, 0.14)	0.244	n.s.	(+) 0.008	n.s.
Relative mass (% total intestine mass)							
Small intestine	46 (43, 48)	< 0.001	− 0.05 (− 0.11, 0.00)	0.069	n.s.	(−) 0.019	n.s.
Caecum^c^	13 (12, 15)	< 0.001	0.18 (0.09, 0.26)	< 0.001	n.s.	n.s.	n.s.
Colon and rectum^b^	38 (35, 41)	< 0.001	0.02 (− 0.07, 0.11)	0.657	n.s.	n.s.	n.s.

Body mass in kilograms.* Plus sign in parentheses* indicates higher values in females, or higher values in formalin-preserved tissues

^a^Results of additional models testing for effects of sex and preservation using ranked data

^b^Residuals were not normally distributed

^c^Body mass was not significant in the ranked-data model

## Discussion

The results of the present study are of relevance to both methodological and biological aspects of digestive tract anatomy, and for compilations of large comparative datasets. The data indicate substantial intraspecific variation in intestinal measurements within mature specimens of *L. catta*. For example, Fig. 4d indicates that at a body mass of 2 kg, the length of the intestinal tract in ring-tailed lemurs may vary by 1 m. In humans, at evidently higher body masses, the documented variation in small intestine length can exceed 4 m (reviewed in Zhou et al. 2020). A large body of literature documents intraspecific variation in intestinal length in rodents due to diet and/or energetic constraints, and intraspecific intestinal length flexibility has been linked to the number of different habitats small rodent species can occupy (reviewed in Naya et al. 2008). For large mammals no corresponding compilations of data exist. To date, variations in digestive anatomy as described for humans, or the lemurs in the present study, remain largely unexplained; however, the variations are of such a magnitude that interobserver error appears a very unlikely cause. In the lemurs studied here, effects of diet or different husbandry conditions also appear unlikely. Thus, the variation remains unexplained. While such variation may not be a systematic problem for large-scale comparisons of intestinal length (Woodall and Skinner 1993; Lavin et al. 2008; Lovegrove 2010), studies exploring quantitative differences between only a few specimens of a few species need to take this variation into account and should include a sufficient number of individuals.

Given the magnitude of this general intraspecific variation, the variation introduced by the use of formalin-fixed material appeared to be of a negligible magnitude in the present study. The material that was compared had either been frozen at an unknown time (but most likely within 24 h) after death, then thawed and dissected from the mesenteries, or dissected at an unknown time after death and subsequently placed in formalin. Of the two processes, freezing and thawing could be assumed to counteract any potential effect of post-mortem contraction. By contrast, formalin fixation could theoretically have occurred at any stage of post-mortem contraction or relaxation, and therefore, shorter dimensions, on average, could have been expected for this method. However, the opposite was the case, with formalin-fixed specimens showing somewhat longer large intestines (Table 2). One theoretical explanation for this could be the lengthwise opening of the intestines, which would not have affected the length of the smooth-walled small intestine, but may have had an effect on the haustration of the large intestine: when opened, the haustra might not constrain the length of the organ as much as they do in a closed state. Unfortunately, this finding only became evident after the frozen/thawed material had been disposed of, otherwise a comparison of the length of the same material with the intestine closed and opened could have been performed. However, the formalin-fixed large intestines were also heavier—longitudinal cuts should not affect mass measurements—suggesting that this difference between the preservation methods for the large intestine might simply have been due to chance.

Overall, the fact that intraspecific variability was of a magnitude that rendered the effect of preservation secondary suggests that, for large-scale comparisons, data from both conservation methods should be acceptable. It should be noted that our study does not represent an experimental approach to preservation methods, where the same material is measured repeatedly after exposure to different treatments, or material from the same facility exposed to different treatments. Most particularly, the present study did not include intact carcasses preserved in formalin with the gastrointestinal tract in situ. Under that condition, shorter measurements might be expected (Zhou et al. 2020). By contrast, preserving intestinal material in formalin after exenteration (and removal of mesenteries), as done in one of the facilities of the present study, does not appear to lead to a systematic deviation in macroanatomical measures, and therefore represents a suitable way of preserving intestines for long periods of time.

Regardless of the large variation in intestinal measures in mature specimens, the intraspecific allometry, including that of neonates and juveniles, yielded scaling relationships comparable to those previously reported in the literature in interspecific studies (see “Introduction”), in the range of a 0.4–0.5 scaling exponent. A similar, more-than-geometric (or positively allometric) intraspecific scaling of the small and the large intestine across ontogeny was demonstrated in rats (Toloza and Diamond 1992) and mice (Wołczuk et al. 2011). As in the ring-tailed lemurs examined here, the scaling effect was mostly found in the neonate and juvenile stages, and was not evident within mature specimens. The more-than-geometric scaling of intestinal lengths, as explained in the Introduction, appears to be a general feature of mammalian macroanatomy.

In ruminants and possibly other foregut fermenters, the change in proportions of the different GIT sections during the transition from milk-feeding to weaning are very distinct. Indeed, the fermentation compartments increase disproportionately in tissue weight (e.g., Wardrop and Coombe 1960; Godfrey 1961) and, by inference, in volume. In horses, the length proportions of the caecum and proximal colon—which represent the fermentation chambers—similarly increase with age until maturity (Smyth 1988). For the ring-tailed lemurs of the present study, a similar ontogenetic change in GIT proportions linked to a change in diet was not evident in the length measurements.

The scaling of organ tissue masses surprisingly appeared more-than-linear (or, again, showed positive allometry), with 95% confidence intervals of the scaling exponent consistently above 1.00 (Table 3). To our knowledge, no recent comprehensive treatise on interspecific gastrointestinal tissue mass exists. Calder (1996) cites the scaling in 41 mammal species established by Brody (1945) with an exponent of 0.94; using the SE for the exponent given in the original study by Brody (1945), the 95% confidence interval of that exponent includes linearity at 0.85–1.03 (and is nearly identical for the exponent found for birds in that study). In the original data from Navarrete et al. (2011) for 100 mammal species, a similar scaling exponent with a confidence interval of 0.88–0.94 can be calculated, and Prothero (2015) found a scaling exponent of 0.93 in mammals that also excluded linearity in the confidence interval. Why mammalian and avian GIT scaling should be slightly less-than-linear has not been explained so far, and we are also unable to offer an explanation for this. We hypothesize that the more-than-linear scaling found in our data is an intraspecific effect of ontogeny, reflecting the shift from milk feeding to solid food. In the case of the ring-tailed lemur, the natural diet comprises fruits, leaves and other plant parts (e.g., Rasamimanana and Rafidinarivo 1993; Simmen et al. 2006). On the one hand, an increasing tissue mass with age could derive from a disproportionately increased muscle mass as an effect of processing solid material. On the other hand, it could derive in particular from absorptive mucosa development in those compartments (caecum, colon) where fermentative digestion intensifies after the switch to solid food. The enhancing effect of short-chain fatty acids, the main products of microbial fermentation, on gut mucosa development—and hence tissue mass—is well known (e.g., Kripke et al. 1989). Fermentative microbial digestion has been suggested for ring-tailed lemurs (Campbell et al. 2000), and was demonstrated by the measurement of short-chain fatty acids in the faeces of captive specimens (McKenney et al. 2018). Correspondingly, the scaling exponent of tissue mass was highest for the caecum, followed by the colon and rectum, whereas the small intestine only scaled slightly higher than linearly, and the stomach scaling was linear (Table 3). A shift in the faecal microbiome with the transition from milk-feeding to weaning has been demonstrated in lemurs, including ring-tailed lemurs (McKenney et al. 2018), which would be expected to parallel the increase in tissue mass.

No evidence was found in the present study for the presence of a caecal appendix in ring-tailed lemurs. A review of the primate appendix by Fisher (2000) did not include the ring-tailed lemur as either a species with or without an appendix, and neither Campbell et al. (2000) nor McGrosky et al. (2019b) reported evidence for an appendix in ring-tailed lemurs. The external appearance of the caecum of some individuals included an apparent narrowing of the caecal apex that created the impression of an appendix (Fig. 2), but neither thickening of the mucosa nor macroscopic appearance of lymphatic tissue were evident (Fig. 3). A recent description of the gastrointestinal anatomy of another lemur species, *Eulemur coronatus*, also did not suggest the presence of an appendix (Schwitzer 2009), although the species is among those for which an appendix is assumed in the literature (Fisher 2000; Smith et al. 2013, 2017). A more detailed histological study of the putative appendices of lemur species might be interesting.

To conclude, the present study emphasizes that, even though comparative studies might have to work with species averages, one should not forget that biological features may show a large range of interindividual variability that does not necessarily lend itself to easy explanation. Across the different ontogenetic stages of *L. catta* examined in the present study, there is an indication both for a similar more-than-geometric (or positively allometric) scaling of gut length, and for a relative increase in tissue mass of those sections of the gastrointestinal tract where fermentation occurs with the dietary shift from milk to solid food, the caecum and the colon. Experimental approaches to organ preservation notwithstanding, the present study suggests that measurements of material preserved frozen—either as whole carcasses, or after dissection of the carcass—or of material preserved in formalin after dissection, can be equally used for a comparative study. We hope that the ease with which macroanatomical measurements can be taken, and the ubiquitous opportunity for this in the form of zoological institutions where animals live and die, will facilitate the establishment of updated datasets that can be used to test hypotheses ranging from those on physiology to evolutionary history.

## Electronic supplementary material

Below is the link to the electronic supplementary material.

Supplementary file1 (XLSX 17 KB)

## Data Availability

The data set supporting this article has been uploaded as part of the supplementary material.
